# Biofilm reactors for industrial bioconversion processes: employing potential of enhanced reaction rates

**DOI:** 10.1186/1475-2859-4-24

**Published:** 2005-08-25

**Authors:** Nasib Qureshi, Bassam A Annous, Thaddeus C Ezeji, Patrick Karcher, Ian S Maddox

**Affiliations:** 1 National Center for Agricultural Utilization Research, United States Department of Agriculture**, Agricultural Research Service, Fermentation Biotechnology Unit, 1815 N University Street, Peoria, IL 61604, USA; 2 Eastern Regional Research Center, United States Department of Agriculture, Agricultural Research Service, 600E Mermaid Lane, Wyndmoor, PA 19038, USA; 3 University of Illinois, Biotechnology & Bioengineering Group, Department of Food Science & Human Nutrition, 1207 W Gregory Drive, Urbana, IL 61801, USA; 4 Massey Univesity, Institute of Engineering & Technology, Palmerston North, New Zealand

## Abstract

This article describes the use of biofilm reactors for the production of various chemicals by fermentation and wastewater treatment. Biofilm formation is a natural process where microbial cells attach to the support (adsorbent) or form flocs/aggregates (also called granules) without use of chemicals and form thick layers of cells known as "biofilms." As a result of biofilm formation, cell densities in the reactor increase and cell concentrations as high as 74 gL^-1 ^can be achieved. The reactor configurations can be as simple as a batch reactor, continuous stirred tank reactor (CSTR), packed bed reactor (PBR), fluidized bed reactor (FBR), airlift reactor (ALR), upflow anaerobic sludge blanket (UASB) reactor, or any other suitable configuration. In UASB granular biofilm particles are used. This article demonstrates that reactor productivities in these reactors have been superior to any other reactor types. This article describes production of ethanol, butanol, lactic acid, acetic acid/vinegar, succinic acid, and fumaric acid in addition to wastewater treatment in the biofilm reactors. As the title suggests, biofilm reactors have high potential to be employed in biotechnology/bioconversion industry for viable economic reasons. In this article, various reactor types have been compared for the above bioconversion processes.

## Introduction

Biochemical reactors play an important role in the biochemical industry as the rate of reaction, ease, and length of reactor operation affect reactor productivities and hence process economics [1,2]. In order to employ a most appropriate reactor for an industrial operation, reaction rate should be high and the reactor configuration should be simple. Under optimized parameters such as pH, temperature, substrate, and medium components, reaction rate can be increased by increasing cell mass concentration in the reactor. There are two methods commonly used for increasing cell mass concentration inside the reactor; first, use of a permeable membrane to retain cells; and the other, use of immobilized cell technique. Membrane reactors allow passing of liquid, substrate, and product out of the reactor while retaining the cells. In these reactors, high cell concentrations can be achieved [3]. Unfortunately, for some processes such as waste water treatment, these reactors are not preferred due to their high cost and problems with fouling. Other processes where the relatively high cost of these reactors does not allow their use include production of large volume, low cost chemicals such as vinegar or acetic acid.

Other types of reactors that offer high reaction rates are immobilized cell reactors [4]. In these reactors, high cell concentrations are achieved by fixing them on various supports. Cells can be immobilized by three different techniques; namely, adsorption, entrapment, and covalent bond formation. Entrapment and covalent bond formation require use of chemicals that add to the cost of production and perhaps restrict further propagation or increase in cell concentration inside the reactor. The third technique is of natural origin as cells "adsorb/and adhere" to the support naturally and firmly [4-6]. This technique is called "adsorption" and has been used extensively in the literature to adsorb microbial cells. Table 1 shows a comparison of these techniques with the membrane reactors. It should be noted that some microbial cells leach out from immobilized cell reactors which require separation (leached out cells in reactor effluent) prior to product removal possibly by centrifugation.

**Table 1:** A comparison of different types of reactors with biofilm reactors

**Reactor Type**	**Comments**
**Membrane reactor**	
Advantages	High productivities, high cell concentration can be achieved inside the reactor, clear permeates for further separation
Disadvantages	Fouling with cells, cost prohibits their use in low cost large volume chemical production
**Immobilized cell reactors**	
**Covalent bond formation**	
Advantages	High cell concentration may be achieved, high productivity
Disadvantages	Cell growth inside matrix may be restricted, cells leach out of the matrix and hence centrifugation of effluent may be required, chemical may affect the cells
**Entrapment**	
Advantages	High cell concentration may be achieved, high productivity
Disadvantages	Matrix often starts disintegration with time, cells leach out of matrix, centrifugation of reactor effluents is required for further separation
**Biofilm**	
Advantages	Comparatively high reactor productivities and high cell concentrations are achieved, reactors run longer and are economic to operate
Disadvantages	Effluent centrifugation is required

In addition to being a natural process, adsorption can be performed in place, and economical adsorbents are available. Additionally, these reactors are simple in concept and construction and the immobilization process is economical. Adsorbed cells form cell layers on the support and cell mass grows inside the reactor over time [7]. These layers of cells are called "biofilms." Biofilms can be used in various types of reactors such as continuous stirred tank reactors (CSTRs), packed bed reactors (PBRs), fluidized bed reactors (FBRs), airlift reactors (ALRs), upflow anaerobic sludge blanket (UASB) reactors, and expanded granular sludge bed (EGSB) reactors etc. [4,7-11]. In these reactors, reaction rates are usually high as compared to the other types of reactors. On the laboratory, pilot plant, and industrial scale (some), these reactors have been very successful and examples include waste water treatment [12] and vinegar or acetic acid [13] production. In addition to these, other processes that have employed these biofilm reactors include ethanol, butanol, lactic acid, fumaric acid, and succinic acid production. Since they offer high reaction rates and are economical, this review becomes their subject matter. In the authors' view, this natural process of biofilm formation can be employed to economize production of various chemicals by fermentation on a large scale [13]. In biofilm reactors, cell concentrations as high as 74 gL^-1 ^can be achieved [7]. In addition, the cell layers in bioparticles become highly active, thus contributing to the high reactor productivities. Within fluidized bed reactors, the biofilm particles are of various shapes (including spherical and irregular shapes) and these reactors can be operated for long periods of time. The amount of adsorbent that is used in these reactors is low, which also reduces the cost of the cell support. In biofilm reactors, reactor configurations can vary from a simple packed bed reactor to fluidized bed, UASB, and airlift reactors as described in this article.

## Biofilm Formation

### Various types of biofilms

In nature, biofilms exist primarily as complex multi-species communities of bacteria in which each species fills an ecological niche within the biofilm depending on its metabolism and morphology [14]. The nature of mixed culture biofilms is dependent on which species are present and what role each species fills. For instance, a single species may utilize anaerobic fermentation deep within one biofilm in one environment, but may utilize an aerobic metabolism in another environment in the presence of different neighboring biofilm species. Multi-species biofilms are important clinically as well as industrially. Clinically, biofilms are important as the source of persistent infections. They are responsible for dental caries and nosocomial infections, as well as a variety of other infections and diseases [15]. Industrially, biofilms are detrimental in many cases and beneficial in many others. For instance, natural biofilms can reduce heat transfer in heat exchangers and cooling towers [16], foul reverse osmosis membranes [17], and contaminate food processing equipment [18]. Multi-species biofilms are used industrially to achieve several aims including the treatment of wastewater for removal of organics [19,20] and heavy metals [21]. The presence of multiple species allows for the treatment of waste streams that are diverse in composition and that fluctuate in component concentration.

Single species biofilm are used to produce industrially important chemicals [22,23]. Such biofilms can exist in some situations and are important industrially, although in nature they are not the norm. For example, in nature, an immature biofilm that is resultant from the attachment and growth of a single cell may exist as a single species biofilm before incorporating other species. For chemical production, single species biofilms are important because they allow for control and maximization of desired products. In this case, a single species is inoculated into a sterile environment and allowed to form a biofilm before being used to produce a particular chemical product.

In industrial applications including wastewater treatment, usually two types of biofilms are employed, namely, biofilms that grow onto supports such as charcoal, resin, bonechar, concrete, clay brick, or sand particles, and biofilms that are formed as a result of flocs and aggregate formation. On the above supports, biomass grows all around the particles and the size of the biofilm particles grows with time usually to several mm in diameter. The density of the support particles is usually higher than the fermentation broth and for this reason bioparticles tend to remain in the lower section of the reactor. Another type of biofilm is where no support is used and cells form biomass granules and flocs that also grow in size with time. This type of biofilm is called granular biofilm and the reactor where this biofilm is used is called granular biofilm reactor. Granule formation may take from several weeks to several months. The cells produce extracellular polymeric substances (EPS) that binds the cells firmly in the form of flocs and aggregates. The most commonly used bioreactors that fall in this category are upflow anaerobic sludge blanket (UASB) reactors that are used to treat domestic and industrial wastewater anaerobically. Sponza [24] examined anaerobic granulation process in a UASB to remove tetrachloroethylene. In some cases expanded bed biofilm reactors have been used with granular biofilm particles that are called expanded granular sludge bed (EGSB) reactors.

### Mechanism of biofilm formation

A biofilm is defined as a structured community of bacterial cells enclosed in a self-produced polymeric matrix and adherent to an inert or living surface [15]. In general, there are four stages to the development of a mature biofilm: initial attachment, irreversible attachment by the production of extracellular polymeric substances (EPS), early development, and maturation of biofilm architecture [14].

The life of a biofilm starts with the planktonic or free floating cell. In order for a planktonic cell to attach to a surface, it must first interact with the surface. Surfaces immersed in an aqueous solution usually acquire a surface charge which attracts and concentrates inorganic solutes, and charged or highly polar organic molecules. The concentration of cations, glycoproteins, proteins, and organic molecules at the surface can provide a relatively nutritious zone for bacteria compared to the bulk aqueous environment [25]. In addition, fluid flow in the boundary region near the surface can be considered negligible which allows bacteria to approach the surface. Once near the surface, it will either approach the surface by Brownian motion or move by chemotaxis towards the surface in response to the chemical concentration gradient [25]. When at the interface, the cell will form a temporary association with the surface or microbes already present on the surface [26].

After initial association with the surface, a planktonic bacterial cell can dissociate from the surface and resume the planktonic state or become irreversibly attached to the surface. Irreversible attachment involves the production of EPS. EPS serves to bind the cell to the surface and to protect it from the surrounding environment. EPS can be composed of polysaccharides, proteins, nucleic acids, or phospholipids. A common EPS produced by bacterial cells in biofilms is the exopolysaccharide alginate. In biofilm associated cells of *Pseudomonas aeruginosa*, transcription of *algC*, the gene involved in alginate production, was fourfold that in planktonic cells [27]. EPS provide protection to biofilm cells by providing a diffusive barrier to any toxic compounds that could harm the cells as well as a barrier to phagocytes and bacteriocides. The EPS can also represent a barrier to nutrients necessary for cell growth. Cells in the interior of a biofilm often show a much reduced rate of growth and cell division rate may be near zero [26,28]. The reduced growth rate is itself protective because uptake of toxic substances is also reduced. The presence of the EPS matrix may also serve as a spatial restrictor of cell growth and division.

Water and nutrient diffusion into the interior of a biofilm is highly limited. As biofilms mature, water channels can develop that allow water and nutrient access deeper into the biofilm. These channels partially relieve the diffusion limitation within the biofilm. The architecture of the biofilm develops in response to shear forces. In low shear environments, biofilms can form as thick mushroom-like masses. In high shear environments, biofilms may be flatter or form long strands [29].

A final stage that may occur in the life of a biofilm is reversion of part of the cells to the planktonic state. When cells living in biofilm take up nutrients, they channel much of that energy towards production of EPS rather than to cell growth and division. When nutrients become scarce, cells must escape the EPS matrix or be trapped in an unfavorable environment. Biofilm associated cells are able to produce enzymes capable of breaking down the EPS matrix in times of nutrient starvation. *Pseudomonas fluorescens *is able to produce an exopolysaccharide lyase under starvation conditions [30]. The enzyme serves not only to break down the polysaccharide matrix allowing cells to find nutrients elsewhere, but the degraded EPS can often be used as a food source for the nutrient deprived cells. In addition to cell detachment due to starvation or nutrient deficiency, there are other detachment processes such as abrasion, shear stress, sloughing and grazing. Providing detailed accounts of these processes is considered beyond the scope of this article.

### Factors enhancing biofilm formation

Several parameters affect how quickly biofilms form and mature, including surface, cellular, and environmental factors. The surface onto which cells will attach has an important impact on biofilm formation. Rough surfaces tend to enhance biofilm formation [31]. Shear forces are lower near a rough surface, and there is a larger surface area to which cells can adhere. Porous materials also work well for biofilm formation. Shear forces are very low inside pores even under conditions where bulk fluid velocity is high. Pores provide a protected environment for cells to attach and grow. Porous materials such as brick and bonechar have been used to immobilize *Clostridium *cells used in biofilm reactors [22,32]. Biofilm formation also tends to increase with the hydrophobicity of the surface material [28]. Biofilms form much more rapidly on Teflon and other plastics than glass or metal. Possibly this is due to differences in hydrophobicity of the surfaces and ionic charges [28].

The amount of nutrients present in the medium can affect the rate of biofilm formation. Biofilms tend to form more readily in the presence of ample nutrients [33]. One function of the biofilm is to anchor cells in a friendly, nutrient rich environment. Phosphorus is a particularly important nutrient. Cells saturated with phosphate have a higher tendency to flocculate and adhere due to their increased hydrophobicity, while those cells depleted in phosphate are more hydrophilic and less likely to adhere [34].

Temperature can have an effect on biofilm formation. Temperatures at the high end of a culture's growth range can enhance biofilm formation. Depending upon the species involved, high temperature increases the rate of cell growth, EPS production, and surface adhesion, all of which enhance biofilm formation [25].

Cellular factors may affect biofilm formation. A hydrophobic cell will be more able to overcome the initial electrostatic repulsion with the solid surface and adhere more readily. The presence of fimbriae, proteinaceous bacterial appendages high in hydrophobic amino acids, can increase cell surface hydrophobicity [28,35]. Flagellated cells show increased ability to attach to surfaces. Flagellar motility may serve to overcome initial electrostatic surface repulsion.

### Calculations and data presentation

In a continuous process, productivity (gL^-1^h^-1^) is calculated as the product concentration in gL^-1 ^liquid multiplied by the dilution rate (h^-1^). In a batch process, productivity is calculated as the product concentration in gL^-1 ^liquid divided by the fermentation time (h). Specific productivity (h^-1^) is calculated as productivity (gL^-1^h^-1^) divided by cell or protein concentration (gL^-1^). Dilution rate (feed flow per reactor volume per h) can be based on total volume of the continuous reactor or void volume. In fully or partially packed bed reactors, void volume is total reactor volume minus the volume occupied by the cell support. For a particular flow rate, dilution rate based on void volume is higher than based on the total reactor volume. In this article, reactor productivities based on both total reactor volume and void volume have been reported as mentioned by the different authors. The reader is advised that it is difficult to correlate/compare the two productivities (based on total reactor volume or void volume) unless void volume fraction (void volume/total volume) is given along with the flow/feed rate. Residence time (h) in the reactor can be calculated by inversing the dilution rate (h^-1^).

### Types of biofilm reactors

Biofilm reactors can be assembled in a number of configurations including batch, continuous stirred tank (CSTR; including agitating continuous reactors, and rotary continuous reactors), packed bed (PBR), trickling bed (TBR), fluidized bed (FBR), airlift reactors (ALR), upflow anaerobic sludge blanket (UASB), and expanded bed reactors. The operation of these reactors changes from reactor to reactor. In a batch biofilm reactor, the immobilized cells have to be utilized for repeated batches. However, it is likely that during the late stationary phase of chemical production the culture would experience inhibition thus reducing productivity. Also, in a batch reactor, productivity would be reduced due to downtime necessary to fill and empty the reactor. If the reactor is packed with biofilm particles, some cells may die or become inactive due to lack of feed during emptying and filling of the reactor. As a result, it is viewed that batch reactors are not practical for biofilms.

In a CSTR feed medium is fed to the reactor and product is withdrawn at the same rate as feed. They are stirred using a mechanical device such as impeller. CSTRs cannot be packed with the adsorbent support covered by biofilms as no agitation can be provided in that case. However, they can be used if fibrous bed support is used for adsorption of cells. In that case, cells can grow and form a biofilm on the fibrous bed. In such a case agitation can be provided. This type of system was used for the production of butanol [36] and lactic acid [37] in continuous operation with a constant feed and a constant effluent from the reactor. In some cases, there may be excessive growth on the surface of the fibrous bed, and the cell layers may be sheared off the support. This type of fibrous bed biofilm CSTRs are called as agitating continuous reactors. Another type of CSTRs called rotating CSTRs have same length/diameter (L/D) ratio as in CSTRs. The rotating CSTRs are placed horizontally (lengthwise) and are rotated along the horizontal axis.

PBRs are different types of reactors as they are packed with suitable support material followed by inoculation with the culture to form biofilm. The reactor is supplied with a feed that is not deficient in nutrients. Depending on the culture, nutrients, and support, biofilm formation may take a few to several days. Such reactors are usually fed at the bottom, thus getting product at the top of the reactor. However, these reactors are prone to blockade due to excessive cell growth. In *C. acetobutylicum/C. beijerinckii *biofilm packed bed reactors, reaction rates up to 45 times that of the batch (control) reactors have been obtained [38,39].

TBRs are different from PBRs as they (TBRs) are fed at the top of the reactor thus obtaining product at the bottom. However, in such reactors some of the biofilms may not get sufficient feed thus affecting reactor efficiency/productivity adversely. Also, in gaseous fermentations gas may occupy significant space in the reactor and may form stagnant pockets. This also may affect the efficiency of the reactor. In anaerobic waste water treatment and acetic acid production, these reactors have been used at large scale successfully.

FBRs have played a successful role in the degradation of toxic phenolic chemicals [40-43] and butanol production [39,44]. In these reactors, cell growth occurs around the adsorbent particles. Formation of active biofilms around the particles and accumulation of sufficient biomass in the reactor may take from 2 to 4 weeks. A major advantage in these reactors is that they can be operated for much longer periods than PBR or CSTRs (with fibrous bed). These reactors do not block due to excessive growth. In these reactors butanol production was increased by approximately 40–50 times that of the batch reactors. These reactors have been operated successfully for longer than 4 months in continuous operation (Unpublished data, Qureshi and Maddox).

Airlift reactors contain two concentric tubes, a riser (an inner tube) and a downcomer (an outer tube). In these reactors, mixing is achieved by circulating essentially air at the bottom of the reactor. As a result of force applied by the air (at the bottom of the inner tube), the liquid in the inner tube moves up which then overflows (the inner tube) downward thus creating eddies to mix the liquid. In some of the airlift reactors downcomer is replaced with an external loop to circulate fermentation broth. Such reactors where air is replaced by an anaerobic gas are called gaslift reactors.

**Figure 1:**
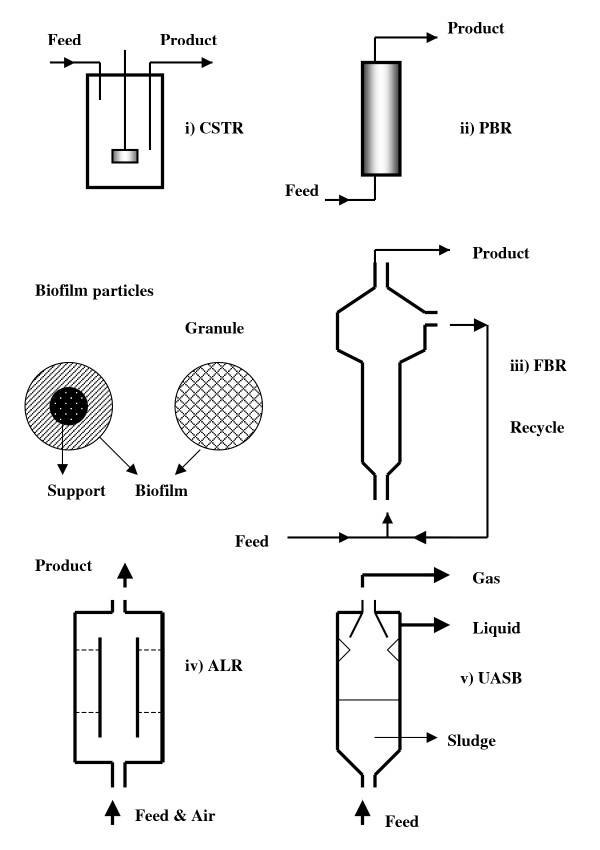
Schematic diagrams of various types of biofilm reactors and biofilm particles.

Upflow anaerobic sludge blanket (UASB) reactors (contain granular biofilm particles) are used for anaerobic treatment of wastewater/industrial effluents. As the name suggests, the flow in these reactors is in upward direction. At the top of the reactor provisions are made for gas/es to escape and sludge particles to settle to the bottom part of the reactor. Reactor effluent is removed from the top of the reactor. UASB reactor was developed by Lettinga et al. [9]. Fig. 1 shows a schematic diagram of various reactors and biofilm particles.

### Biofilm reactors in biological wastewater treatment

The application of biofilm technology in wastewater treatment originated from the industrial operation of trickling filters in the early 1880s in Wales, Great Britain [45]. Biofilm processes in wastewater treatment can be divided into two categories: namely (1) the fixed-medium systems where the biofilm media are static in the reactors and the biological reactions take place in the biofilm developed on the static media, and (2) the moving-medium systems where the biofilm media are kept continually moving by means of mechanical, hydraulic, or air forces [46]. The moving-medium systems include rotating biological contactors, moving-bed biofilm reactors, vertically moving biofilm reactors, and fluidized bed biofilm reactors; while the fixed-medium systems include trickling filters and biological aerated filters [46]. Rotating biological contactors (RBC) have been widely used in biological treatment of wastewater for reducing chemical oxygen demand (COD)/biological oxygen demand (BOD) [47,48] and nitrification/denitrification purposes [48,49]. Rotating biological contactors treat wastewater streams using a thin biofilm of aerobic microorganisms on rotating cylinders or biodiscs. The rate of rotation is selected to provide optimum contact of the waste stream with the biofilm for efficient oxygen transfer and bioactivity.

It should be noted that before a biofilm-based treatment system is to be considered for the treatment of wastewater, it is necessary to determine whether the naturally occurring microorganisms are able to produce biofilms, while simultaneously reducing the COD of the wastewater, or if there is a need to inoculate the reactor with external bacterial strains. The most commonly used rotating biofilm contactor is the rotating biodisc and its various modifications [48]. For the treatment of high strength wastewater, gentle aeration of the liquid phase has been shown to improve the COD reduction of the system by about 40% [50]. In a related study, Kargi and Eker [48] have shown that a rotating-perforated-tube biofilm reactor is effective in COD removal from synthetic wastewater composed of diluted molasses, urea, KH_2_PO_4_, and MgSO_4_. The liquid phase in the tank was not aerated, (the total biofilm surface area (A) was 1.34 m^2^), and the rotation speed of the tubes was 5 rpm [48].

In some instances, thermophilic aerobic systems have been employed to biodegrade the wastewaters of high strength, and tremendous COD reductions have been reported in both laboratory and pilot scale experiments [51,52]. However, the thermophilic systems exhibited poor bacterial flocculation characteristics due to the dispersed growing microorganisms (no biofilm formation) which made bacterial separation from the treated effluent difficult [53,54].

The fluidized bed biofilm reactors (FBBR; also called as FBR) (in which particles move up and down within the expanded bed in the well defined zone of the reactor) have been used for more than two decades for treating industrial wastewater [55,56]. Immobilized bacterial systems configured as fluidized bed biofilm reactors (FBBRs) offer some technical advantages. Since chemical wastes are injected into the recycle, toxic chemicals are immediately diluted, which make the microorganisms more resistant to direct chemical toxicity than many conventional treatment systems. In addition, since FBBRs are usually oxygenated by supplying air into the recycle loop, a high level of microbial activity may be supported with minimal air stripping of volatile chemicals.

Jesis and Owen [57] studied FBBR, and they found that the use of small, fluidized media enabled the FBBR to retain high biomass concentrations and, thereby, operate at significantly reduced hydraulic retention times. In pilot scale operations carried out by Jesis and Owen [57], they reported that when the volatile solid concentrations were between 30,000 to 40,000 mgL^-1 ^during denitrification operation, 99% of influent nitrates could be removed under hydraulic retention times as low as 6 min. In a related study, Rabah and Dahab [56] during their evaluation of the use of fluidized-bed biofilm reactors for nitrate removal concluded that the FBBR system is capable of handling an exceptionally high nitrate nitrogen concentration of 1000 mg(N)L^-1 ^with very high removal efficiency, up to 99.8%. The authors noted that higher denitrification rates can be achieved at relatively low superficial velocities because it is possible to maintain high biomass concentration at lower velocities. However, there is a minimum practical velocity below which agglomeration of media would occur and the process may fail [56]. The efficiency of the FBBR can be up to 10 times greater than that of the activated sludge system and typically occupies 10% of the space required by stirred tank reactors of similar capacities [56]. Higher biomass concentration in the FBBRs (40, 000 mgL^-1^) compared to 3000 mgL^-1 ^in the activated sludge have been shown to be the reason for the greater efficiency [58].

Anaerobic treatment of wastewater in fluidized bed reactors is another area that has been studied extensively [59]. In this article, Iza [59] presented theoretical basis for design and operation of a fluidized bed reactor for anaerobic treatment of wastewater. The anaerobic fluidized bed technology offers a number of advantages for treating wastewater including high concentration of biomass attached to the dense support that makes it possible to operate them at high dilution rate without cell washout. In these reactors no plugging, gas hold-up or channeling occurs.

Prior to the development of UASB, interest in anaerobic treatment systems in wastewater treatment was scarce [11]. Interestingly, the development of UASB saw a significant increase in anaerobic removal of various chemicals from the wastewater using these reactors. The examples include anaerobic removal of pentachlorophenol [60], nitrogen removal [61], dechlorination using *Dehalospirillum multivorns *[62], anaerobic treatment of municipal solid leachate [63], and starch degradation [64]. More studies on this subject have been reviewed in Veeresh et al., [42] (anaerobic treatment of phenol and cresols in UASB reactors). The success of the UASB concept relies on the establishment of a dense sludge bed in the bottom of the reactor which is usually a result of microbial growth and incoming sludge.

Seghezzo et al., [11] reported that in a pilot plant UASB reactor, internal mixing was not optimal for treating sewage (4–20°C) which produced dead space and hence reduced process efficiency. In order to improve the process efficiency, an adequate influent distribution was sought. The use of effluent recirculation in combination with a taller reactor (a larger height to diameter ratio) resulted in the expanded granular sludge bed (EGSB). Usually expended bed reactors, as opposed to EGSB, have biofilm that is adsorbed onto support particles. An example of expanded bed reactor is that of Tsuno et al., [43] who degraded pentachlorophenol (PCP) in a biological expanded-bed reactor anaerobically. In this reactor the granular activated carbon was used as a support.

Generally, total nitrogen removal from domestic or industrial wastewater streams is achieved in two steps: microbial nitrification of ammonium (aerobic process) followed by denitrification (anaerobic process) or reduction of formed nitrate to nitrogen. This conventional method employs a sequence of aerobic and anoxic processes in order to provide the two different environmental conditions [65]. However, studies have shown that these two important steps can occur simultaneously in one reactor in a process called simultaneous nitrification and denitrification (SND). In SND process, nitrification is restricted to the outer oxic zone of formed microbial flocs, whereas denitrification occurs predominantly in the inner anoxic zones [65]. To test the hypothesis that SND is a physical phenomenon, Pochana and Keller [66] carried out experiments to determine the effect of floc size on SND. Typical floc sizes as measured in their experiments were 50 – 110 μm, which is large. Such large floc sizes could create an anoxic zone inside the flocs leading to denitrification. Pochana and Keller [66] concluded that a substantial anoxic mass fraction exists in the center of the biomass floc resulting from an oxygen diffusion limitation into the floc. The rates of nitrogen removal (coupled nitrification-denitrification process) or productivities/specific productivities are shown in Table 2. The sequencing batch and single activated sludge flocs reactors require some O_2 _to effect nitrification (NO_3_^-^/NO_2_^- ^generation), which is the precursor for denitrification process.

**Table 2:** Productivities of different reactors employed for nitrification and denitrification/during the pretreatment of domestic or industrial wastewater streams.

**Reactor Type**	**Removal rates (Productivities or specific productivities)**		**Reference**
		
	**Nitrification**	**Denitrification**	
Activated sludge flocs (Single reactor)	24 μmol N g MLSS^-1 ^h^-1^	6 μmol N g MLSS^-1 ^h^-1^	[65]
Sequencing batch	19 mg NH_3 _L^-1^h^-1^	13.5 mg N L^-1^h^-1^	[66]
Chemostat (Continuous)	5.6 μmol NH_3 _h^-1 ^mg protein^-1^	5.6 μmol N h^-1 ^mg protein^-1^	[67]
Chemostat (Continuous)	2.6 μmol NH_3 _h^-1 ^mg protein^-1^	4.1 μmol N h^-1 ^mg protein^-1^	[68]
Continuous	250 μmol NH_3 _L^-1^h^-1^	400 μmol N L^-1^h^-1^	[69]

MLSS = Mixed liquor suspended solid

In contrast to previous view that denitrification occurs under anaerobic conditions [67] it (denitrification) has been shown to occur under aerobic conditions with a wide range of bacteria [68,70]. Robertson and Kuenen [71] observed that under fully aerobic conditions, *Thiosphaera pantotropha *carries out the following reactions sequentially and simultaneously, in the presence of a suitable electron donor such as acetate.



This implies that the organism can convert ammonia into nitrogen gas without intermediary accumulation of nitrite. Investigating the reason why this organism denitrifies under aerobic conditions, Robertson and Kuenen [72] demonstrated that denitrifying enzymes were present even when the organism was growing aerobically without nitrate.

The last decade has witnessed an increased interest in membrane bioreactors for wastewater treatment [73,74]. The membrane-aerated biofilm reactor (MABR), whereby the biomass is immobilized on membranes through which oxygen is supplied seems to be the most promising design. Results from studies with MABRs have been reported for the degradation of phenol [40], chlorophenols [41], xylene [75], and ammonia [76]. Stripping losses of volatile organic compounds are minimized, and the oxygen partial pressure in the gas compartment allows easy control of oxygen penetration into the biofilm; the dissolved oxygen gradient across the membrane and the biofilm offers an ideal environment for aerobic strains, and foaming due to surfactants can be prevented [74]. For high strength wastewaters, the possibility of enhanced oxygen penetration depths makes MABRs an attractive option for pollutant biodegradation. However, Casey et al. [74] reported that an excessive growth of biofilm is frequently observed. Therefore, biofilm growth should be controlled when operating this reactor. Although a significant amount of work has been performed on the use of biofilm reactors in wastewater treatment, it is beyond the scope of this article to discuss this work in greater detail.

### Biofilms for gas and odor treatment

Traditionally, industrial waste gases have been treated by physico-chemical methods known as adsorption, scrubbing, condensation, and oxidation processes [77]. Biological waste gas treatment is an attractive and environmentally friendly alternative to physico-chemical methods. Industrial waste gases can serve as energy or carbon sources for microbial metabolism. In addition, inorganic waste gases (H_2_S, NH_3_) may be treated directly by employing autotrophic microorganisms which have the ability to utilize CO_2 _as a carbon source for anabolism [78]. Koe and Yang [79] during their evaluation on how to drastically reduce or eliminate the impact of air polluting emissions from wastewater treatment plant suggested that open sources of odorous emissions such as inlet works, primary sedimentation units, aeration tanks, final clarifiers, sludge processing units, and wastewater channels should be covered up and the odorous air be treated before discharging to the ambient atmosphere.

The biofilter, trickling biofilter, and bioscrubber are three major bioreactor designs frequently employed for the treatment of waste gas [78]. A biofilter consists of a filter-bed composed of a carrier (sawdust, compost, dry wastewater sludge, etc.) for the active microorganisms and as nutrient source [77]. Biofilters operate by facilitating the transfer of odorous gas from waste air blown through the biofilters into biofilms around particles of biofilter medium in which bacteria, fungi and other microorganisms are immobilized. On the other hand, waste gas treatment in trickling biofilters involves use of a biological filter continuously fed with a liquid medium and packed with a synthetic carrier on which biofilms grow [77]. Trickling biofiltration has been used, especially outside the United States, for removal of odorous waste gases such as H_2_S [80]. Several species of microorganisms can oxidize hydrogen sulfide to form odorless sulfuric acid. *Thiobacillus thiooxidans *is capable of oxidizing H_2_S at low pH [81]. For effective H_2_S odor control, an ideal habitat for the growth of sulfide-oxidizing bacteria should be created and competing microbes which normally predominate in aerobic treatment processes should be excluded. De Beer et al., [82] demonstrated that the channels surrounding the cell clusters could increase the supply of oxygen and other nutrients to cells within the biofilm, thus relating structure to function [14]. The biofilm structure appears to be largely determined by the production of slime-like matrix of extracellular polymeric substances, which provide the structural support to the biofilm [14]. The structure of biofilms is largely determined by a number of biological factors such as microorganism growth rate, motility, cell signaling, and the production of extracellular polymeric substances. The physical growth environment may also play a significant role in the determination of the biofilm structure [14], and hence the efficiency. However, excessive biofilm development can lead to clogging of the filter-bed of the reactor [78].

Biomass growth and biofilm development can be limited by reducing nutrient supply although this may decrease reactor performance since higher biomass growth shows higher substrate consumption rates [83]. Therefore, it is important to find a balance between excessive biomass growth to prevent biofilter clogging and the odorous gases removal efficiency. Furthermore, waste gases that are characterized by high concentrations of water-soluble pollutants can be treated with bioscrubber. The bioscrubber consists of two reactors. The first reactor is an absorption column where pollutants are absorbed in a liquid phase. The liquid phase goes to the second reactor, which consists of a filter with an activated carbon medium that supports microbial growth. The high bioactivity in the bioscrubber enhances conversion of waste gases into nonhazardous and less odorous compounds. The effluent leaving the bioscrubber can be re-circulated to the absorption column; this technology allows for good gas cleaning when the gaseous pollutants are highly water soluble [77].

Ottengraf [78] reported that the rate of mass transfer of a given compound to be removed or deodorized is determined by the product of the overall mass transfer coefficient, the total contact area in the column, and the average driving force. Therefore, the absorption of a compound will be higher if its concentration in the wastewater is low and its solubility in water is high [78]. The control of operating parameters to the microorganisms in these bioreactors can sometimes be challenging.

## Production of industrial chemicals in biofilm reactors

### Biofilms and biofilm reactors in ethanol production

Bland et al. [84] produced ethanol in an attached film expanded bed bioreactor of *Zymomonas mobilis*. The cells of *Z. mobilis *were adsorbed onto vermiculite and the culture formed an active biofilm. Based on the total volume of the reactor, a productivity of 105 gL^-1^h^-1 ^was obtained at a dilution rate of 3.6 h^-1^. Usually, in a control batch or free cell reactor a productivity of <4 gL^-1^h^-1 ^is achieved. The increased/enhanced productivity reported here is due to the formation of active biofilm onto the adsorbent.

Adsorbed cells of *Saccharomyces cerevisiae *were used in a packed bed continuous bioreactor to produce ethanol from molasses [4]. The cells were immobilized onto a support of natural origin, possibly sugarcane bagasse. It has been reported that the cells were immobilized by natural mode, which is likely to be adsorption. The amount of cells that was adsorbed onto this support was 0.13 gg^-1 ^support. In this biofilm reactor, the authors reported a productivity of 28.6 gL^-1^h^-1 ^as compared to 3.35 gL^-1^h^-1 ^in a free cell continuous process. The dilution rates in the biofilm reactor and free cell continuous system were 0.47 h^-1 ^and 0.65 h^-1^, respectively. Although immobilized cell reactors (such as this biofilm reactor) are typically operated at higher dilution rates than the free cell continuous reactors, it is not clear why the authors used a lower dilution rate in the biofilm reactor. Since carbon utilization for newly growing cells was reduced, product yield was improved as compared to a batch reactor.

Since ion exchange resins have charge on them, bacterial cells can be adsorbed onto the resins thus forming biofilm layers. This concept was employed by Krug and Daugulis [85] to produce ethanol in high productivity reactors using *Z. mobilis*. To find a suitable adsorbent, 10 ion exchange resins, activated carbon, and ceramic chips were examined. A cationic macroreticular resin was shown to be the most efficient adsorbent to immobilize cells of *Zymomonas mobilis*. The immobilized cells were used in a continuous column and 100 gL^-1 ^glucose was fed to the reactor. As a result of formation of biofilm, the reactor productivity was measured at 135.8 gL^-1^h^-1 ^(void volume based productivity, P_dv _= 377.4 gL^-1^h^-1^). The reactor stopped working due to excessive cell growth and plugging after a period of 200 h of operation.

Other reports on ethanol production in biofilm reactors are those of Kunduru and Pometto [86] and Demirici et al. [8]. Kunduru and Pometto [86] studied ethanol production in continuous reactors using biofilm supports of polypropylene or plastic composite. Employing a culture of *Z. mobilis *and a bacterial support of polypropylene, a staggeringly high productivity of 536 gL^-1^h^-1 ^was obtained at a dilution rate of 15.36 h^-1^. In a control free cell fermentation, a productivity of 5 gL^-1^h^-1 ^was obtained at a dilution rate of 0.5 h^-1^. The biofilm reactor was fed from the top, thus collecting product at the bottom of the reactor.

Kunduru and Pometto [86] used another biofilm reactor of *S. cerevisiae *adsorbed onto a plastic composite support and reported a productivity of 76 gL^-1^h^-1 ^at a dilution rate of 2.88 h^-1^. The reactor productivity in a control reactor was 5 gL^-1^h^-1 ^at a dilution rate of 0.5 h^-1^. Unlike the above biofilm reactor, *S. cerevisiae *biofilm reactor was fed at the bottom, and the product was obtained from the top. It is suggested that for a proper comparison both the reactors should have been fed in the same direction.

In order to enhance biofilm formation, Demirici et al. [8] developed a new support material for the growth of *S. cerevisiae*. A mixture of ground soybean hulls (or oat hulls), complex nutrients, and polypropylene was extruded at high temperature into disks and rings. It is likely that heat sensitive nutrients were inactivated during extrusion. Also, polypropylene film may have covered the nutrients, thus making them unavailable to the culture for cell growth. Since no data have been provided on the time period of formation of biofilm or thickness of biofilm, it is difficult to compare this support with other supports.

In a more recent study, Qureshi et al. [87] produced ethanol in a biofilm reactor of genetically engineered *Escherichia coli *from xylose. The biofilm was formed on clay brick particles, and the reactor was operated continuously for 103 days. The reactor was operated at various flow rates, and reactor productivity was found to be improved compared to a free cell batch process. Table 3 compares ethanol productivities obtained in biofilm reactors of various cultures.

**Table 3:** A comparison of production of ethanol in adsorbed cell biofilm reactors

**System/Support**	**Reactor Type**	**Culture**	**Productivity [gL^-1^h^-1^]**	**Reference**
**Biofilm Reactors**				
Resin	Packed bed	*Z. mobilis*	135.8 (P_dT_), 377.4 (P_dv_)	[85]
Vermiculite	Packed bed^d^	*Z. mobilis*	105.0 (P_dT_), 210 (P_dv_)	[84]
Sugarcane bagasse^a^	Packed bed	*S. cerevisiae*	28.6^b^	[4]
Polypropylene	Packed bed^e^	*Z. mobilis*	536^c^	[86]
Plastic composite	Packed bed	*S. cerevisiae*	76^c^	[86]
**Cell Recycle**	CSTR	*Z. mobilis*	200^b^	[88]
**Batch/Continuous suspended cell (Control)**				
Continuous	CSTR	*Z. mobilis*	5.0^b^	[86]
		*S. cerevisiae*	5.0^b^	[86]
Continuous	CSTR	*S. cerevisiae*	3.35^b^	[4]

a: The support was reported as an adsorbent of natural origin (perhaps sugarcane bagasse)

b: Not reported whether based on total reactor volume or void volume

c: Not reported (possibly based on void volume)

d: Cone shaped

e: Trickling packed bed

P_dT _– Productivity based on total reactor volume

P_dv _– Productivity based on reactor void volume

### Biofilms and biofilm reactors for butanol production

Butanol is an important industrial chemical that can be produced from a number of carbohydrates using a number of microbial cultures. Butanol can be used as a fuel and has higher/greater energy content than ethanol. Production of butanol has been investigated in batch, fed-batch, free cell continuous, immobilized cell continuous, and cell recycle continuous reactors [1]. Continuous immobilized cell and cell recycle reactors offer higher productivities than batch and free cell continuous reactors. In addition to achieving a high productivity, a major advantage of immobilized cell technology is that there is no cell washout at high dilution rates.

Adsorption is a technique which does not require any chemicals for cell immobilization and can be easily performed inside the reactor. In order to immobilize cells of *Clostridium acetobutylicum*, the reactor is packed with an adsorption support followed by inoculation with the culture. The adsorption process varies from 2–3 days to weeks depending upon the culture, support, and the reactor. The culture forms cell layers (biofilm) on the support [5,6,22].

An early report of adsorption of cells of *C. acetobutylicum *for the production of butanol was that of Forberg and Haggstrom [5]. These authors used beechwood shavings to adsorb cells. The reactor was fed continuously with a glucose solution (and nutrient dosing). Over a period of time, an active biofilm was formed on the wood shavings, and a reactor productivity as high as 1.53 gL^-1^h^-1 ^was observed (compared to <0.1–0.35 gL^-1^h^-1 ^in control batch fermentation). This work was followed by experiments examining the production of butanol in adsorbed cell biofilm reactor of *C. acetobutylicum *from whey permeate [6]. It should be noted that biofilm formation on this support was quick, and a reactor productivity of 4.5 gL^-1^h^-1 ^was observed, which was superior to any previously reported butanol production system. Following these reports, Welsh et al. [89] investigated the use of a number of adsorption supports for butanol production by *C. acetobutylicum *in batch and continuous systems. The adsorbents used were coke, kaolinite, and Gel White (a montmorillonite clay). Coke was reported to be superior to other supports for adsorption. A maximum concentration of acetone butanol ethanol (ABE) in the effluent of the reactor was reported to be 12 gL^-1 ^at a dilution rate of 0.1 h^-1^, thus resulting in a productivity of 1.2 gL^-1^h^-1^.

**Figure 2:**
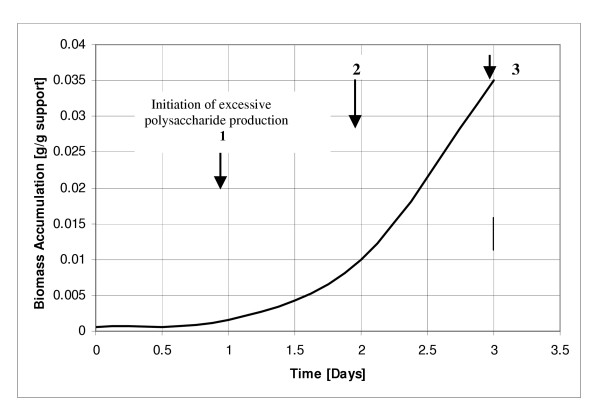
Production of polysaccharide and accumulation of cell mass during the initial 3 days of adsorption of cells of *C. acetobutylicum *onto bonechar for the production of butanol from whey permeate in a packed bed reactor. 1. Initiation of excessive production of polysaccharide; 2. Maximum growth and attachment starts; 3. Biofilms become visible and polysaccharide production continues.

Following above reports, an intensive study was performed on the adsorption of *C. acetobutylicum *on a number of supports and biofilm formation (Table 4, 5). It has been observed that *C. acetobutylicum *and *C. beijerinckii *form visual biofilm layers in 2–4 days (in packed bed reactors), and reactors become productive after 4^th ^day of continuous operation. The techniques of adsorption and reactor operation have been reported previously [6,38,89]. It has been observed that not all the supports are suitable for adsorption (Table 4). It has also been observed that during biofilm formation onto bonechar, the culture produces higher concentration of polysaccharide between day 2 and 4. During this period, up to 2.04 gL^-1 ^polysaccharide production was observed as opposed to 0.95 gL^-1 ^during day 5–30 (Fig. 2). As described in the previous section, the cultures that were used for adsorption for butanol production have flagella, which perhaps help bring the cells closer to the surface of support. In addition, charge on the support and cell is likely to aid in initial adsorption or bringing the cells closer to the support surface.

**Table 4:** Biofilm formation characteristics of *Clostridium acetobutylicum/C. beijerinckii *onto various supports

**Support**	**Characteristics**
Bonechar	*C. acetobutylicum *culture - Adsorption is quick - Biomass layers (biofilms) become visible in 3–4 days time - Between day 2 and 4, the culture produces polysaccharide in high concentrations (2.04 gL^-1 ^broth as compared to 0.95 gL^-1 ^broth from day 5 to 30) - Once initial layers appear, biomass accumulation is quick - Desorption does not occur at high dilution rates - < than 25% cells were desorbed when adsorbed cell particles were agitated at 200–300 rpm (in shake flasks on shaker) at pH 2.7 for 18–24 h at 30°C - During initial stages (2–4 days) the culture produced high concentrations of acids (~6–9 gL^-1^) followed by becoming solventogenic - During solventogenic stages fluctuations in solvent concentrations were less
Glass beads	*C. acetobutylicum *culture - Biomass accumulation takes much longer than bone char - During initial stages (2–4 days) higher amount of polysaccharide production does not occur - Cells do not stick to the support as firmly as onto bonechar - Reactor produces <20% solvents as compared to bonechar adsorbed cells - Reactors are not stable as solvent concentration fluctuates - Cells can easily be washed off
Glass wool, Polypropylene tow, and stainless steel wire balls	*C. acetobutylicum *culture - <20% biomass accumulated than in bonechar packed reactor - Cells do not stick to the support firmly and can be desorbed easily - Reactors are not stable and poor solventogenesis occurred
Clay brick (Ref. 38)	*C. beijerinckii *culture - Cells stick firmly as in case of bonechar and reactors were solventogenic

**Table 5:** Production of solvents in packed bed biofilm reactors of *C. acetobutylicum/C. beijerinckii*

Culture/support	Maximum Solvent [gL^-1^]	Maximum productivity [gL^-1^h^-1^]	Accumulated biomass [gL^-1 ^reactor vol]	Biomass accumulation [gg^-1 ^support]
***C. acetobutylicum***				
Bonechar	9.3 (0.30)	6.50 (1.5)	74.0	0.087
Glass beads	3.0 (0.31)	0.93 (0.31)	65.0	0.044
Glass wool	3.0 (0.10)	0.30 (0.10)	3.1	0.050
Polypropylene tow	2.3 (0.25)	0.58 (0.25)	0.8	-
Stainless steel wire balls	2.0 (0.07)	0.15 (0.07)	1.0	-

***C. beijerinckii***				
Clay brick [Ref 38]	7.9 (2.00)	15.8 (2.00)	73.7	0.093

Numbers in bracket are dilution rates (h^-1^) at which solvent and productivity were obtained

- Values not calculated

**Figure 3:**
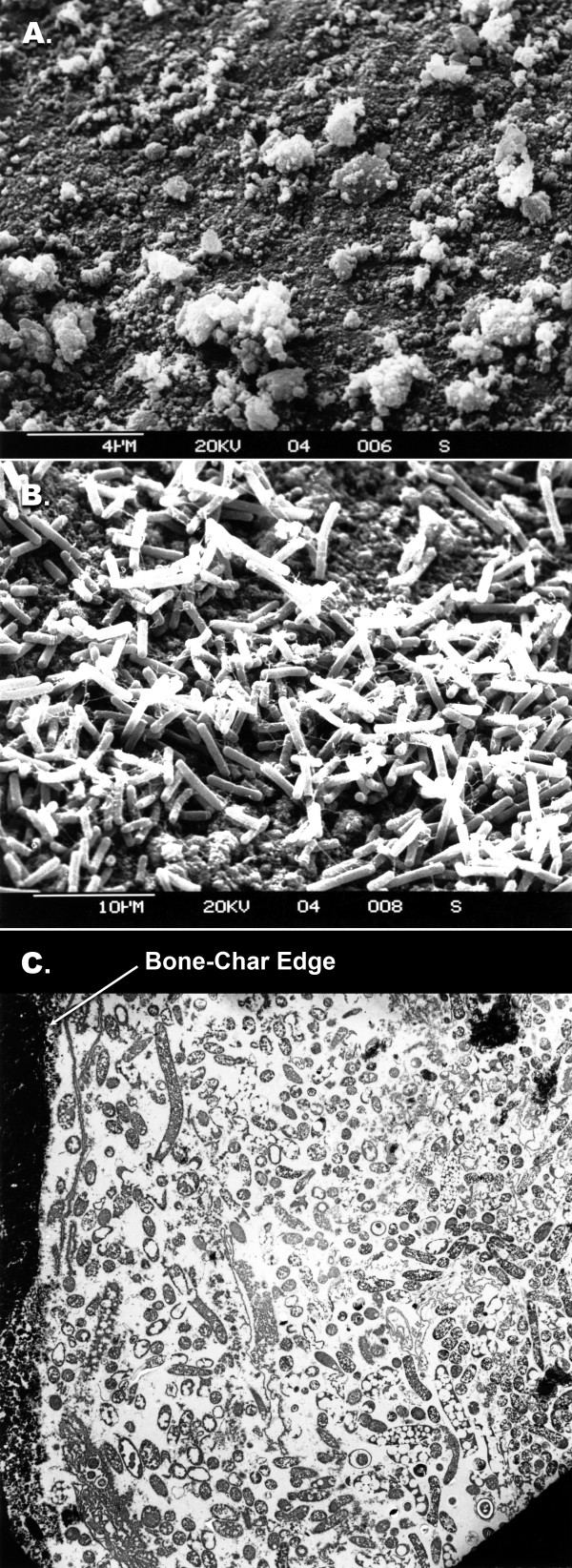
Scanning electron micrograph of adsorbed cells of *C. acetobutylicum *P262 onto bonechar. a) bonechar (magnification 5500); b) adsorbed cells onto bonechar (magnification 2200); c) transmission electron micrograph of adsorbed cells (magnification 2300). Similar figures (3b, c) with different magnification were published previously in the following article: Qureshi N, Paterson AHJ, Maddox IS: **Model for continuous production of solvents from whey permeate in a packed bed reactor using cells of *Clostridium acetobutylicum *immobilized by adsorption onto bonechar. ***Appl Microbiol Biotechnol *1988, **29:**323–328. Figure 3 is reprinted with permission from Springer, Germany (see above article).

Some supports accumulated more cell concentration (*C. acetobutylicum*) and were more solventogenic than the others (Table 5). At this stage, we are not aware what makes some supports better than others for biofilm formation and cell accumulation. From some supports it was easier to wash away the cells while from others such as bonechar and clay brick it was more difficult (Table 4). During our studies on butanol production, it was observed that approximately 0.9–1.0 gL^-1 ^cells were present in the effluent [7,90] of the reactor. We have demonstrated that the cells that are present in the effluent of the reactor are those that grew on the surface of the support, rather than those that grew in liquid medium inside the reactor [90], suggesting that a tremendous amount of activity occurs on the surface of the biofilm in *C. beijerinckii/C. acetobutylicum *cultures. The thickness of biofilm that is formed in *C. acetobutylicum *or *C. beijerinckii *cultures can range from few cell layers to as many as 35 or more. Figure 3 shows adsorbed cells and biofilm formed by *C. acetobutylicum *onto bonechar. Similar observations on biofilm formation were observed for *C. beijerinckii *[38].

**Figure 4:**
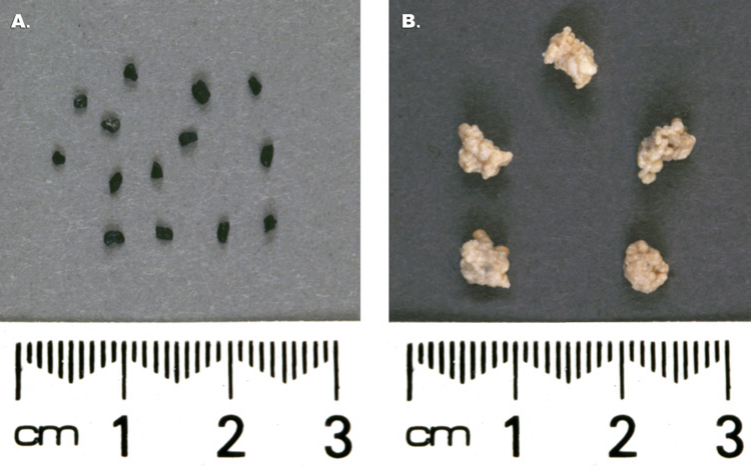
Photographs of biofilm particles of *C. acetobutylicum *P262 used in a fluidized bed reactor for the production of butanol from whey permeate. A) bonechar particles; B) biofilm particles after growth (bonechar particles are covered with biofilm layers).

**Figure 5:**
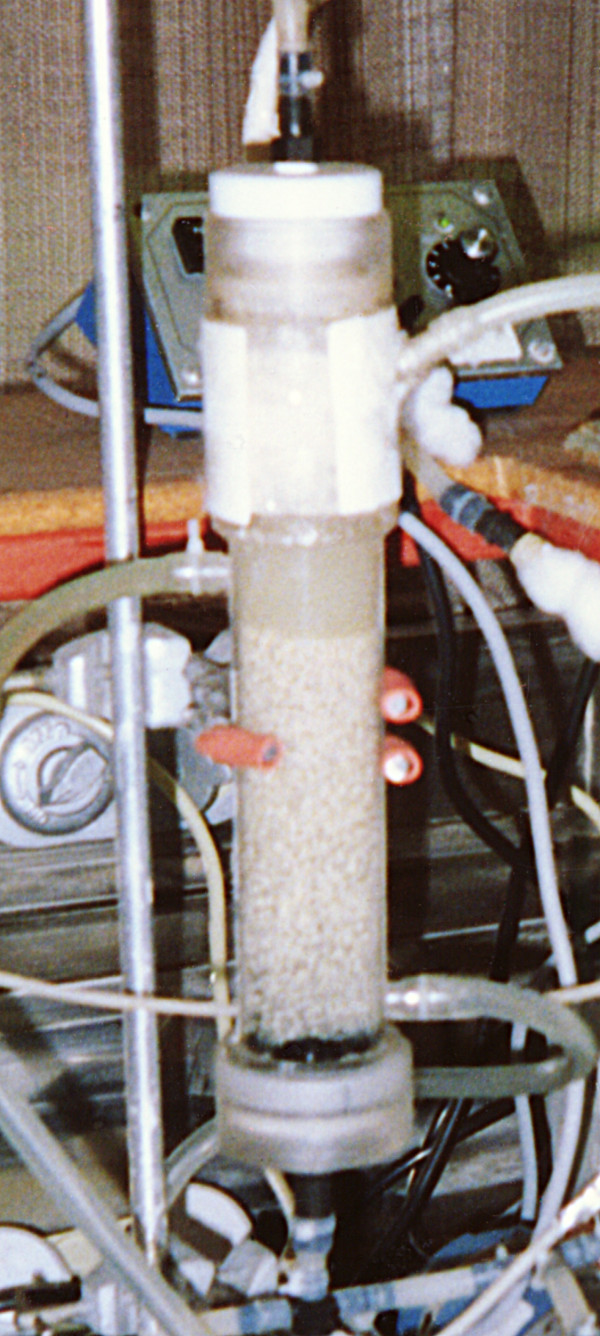
A photograph of a fluidized bed bioreactor (inside volume 450 cm^3^) used to produce butanol from whey permeate using *C. acetobutylicum *P262.

Intensive research has been done on butanol production in various types of reactor systems [1,39,91,92]. The biofilm reactor systems that have been used for butanol production include vertical packed bed reactor (PBR), horizontal PBR, compartmentalized reactor, double series reactors, and FBR. The PBRs and FBRs are different in the sense that FBR is started with support <10% of its volume while packed beds are filled up to 90% of their volume. In packed bed reactors, as cell growth occurs, they are often blocked due to excessive cell growth while in FBRs this does not occur. In FBRs, the bed is fluidized either by recycling fermentation broth, using anaerobic gases (N_2 _or CO_2 _& H_2 _in case butanol fermentation) or air (for other aerobic systems). Cell growth occurs all around the support particles and over a period of time the volume of biofilm particles becomes many fold greater than the support particle (Fig. 3, 4). It should be noted that in FBRs cell growth occurs on the particles in spite of broth's high flow rates [44]. In this fluidized bed reactor liquid flow velocity of the order 40–60 ms^-1 ^was maintained. The reader is advised that despite such a high flow velocity, the culture maintains its growth as a biofilm. We have not calculated the shear rate on the biofilm particles. The reactor was used for the production of butanol from whey permeate in continuous operation for >4 months (unpublished results – Qureshi & Maddox). Newly adsorbed *C. acetobutylicum *cells onto bonechar grow in an exponential manner and accumulation of biomass continues with time. Figure 5 shows a picture of a fluidized bed reactor used for the production of butanol from whey permeate.

Among the various types of reactors used for butanol production, adsorbed cell biofilm reactors (cells adsorbed onto bonechar and clay brick) offered the highest reactor productivities. The reactor productivities that have been achieved in these reactors ranged from 6.5 [39] to 15.8 [38] gL^-1^h^-1 ^(as compared to 0.10–0.38 gL^-1^h^-1 ^in batch reactors). Membrane cell reactors also offer high productivities (6.5 gL^-1^h^-1^) [93,94]; however, biofilm reactors were superior to these reactors (Table 5). Of the various supports tested, bonechar and clay brick were found to be most suitable, and strong biofilms were formed on these supports. *C. acetobutylicum *was adsorbed onto bonechar while *C. beijerinckii *was adsorbed onto clay brick. Attempts were made to desorb the adsorbed cells of *C. acetobutylicum*. In order to achieve this, 100 g bonechar with adsorbed cells (25 days old reactor) was transferred to a 500 mL conical flask. The pH of the solution/reaction mixture was adjusted to 2.7, and the mixture was placed on a rotary shaker at 250 rpm for 18 to 24 h. After this period <30% cells were desorbed from the bonechar.

### Biofilms in 2,3-butanediol production

In an attempt to improve reactor productivity in 2,3-butanediol fermentation, Maddox et al. [23] immobilized cells of *Klebsiella pneumoniae *on to bonechar. The cells of *K. pneumoniae *were adsorbed in a similar manner as *C. acetobutylicum *[6]. During the 2,3-butanediol fermentation, a productivity of 11.7 gL^-1^h^-1 ^was obtained, which was the highest reported productivity. Prior to this work Shazer and Speckman [95] reported a productivity of 1.04 gL^-1^h^-1 ^in 2,3-butanediol fermentation using *Bacillus polymyxa *in a membrane cell reactor. This work clearly demonstrated that bonechar adsorbed cells of *K. pneumoniae *result in superior productivities. Table 6 compares reactor productivity achieved in biofilm reactor as compared to various other reactor types. It should be noted that although high reactor productivity was obtained in the adsorbed cell reactor, cells did not adsorb on to bonechar as strongly as *C. acetobutylicum*. Rather, cells were entrapped in between bonechar particles. However, it is anticipated that there were a significant amount of cells sitting on the surface of bonechar as bonechar surface area was large. At the end of fermentation, it was observed that unlike cells of *C. acetobutylicum*, *K. pneumoniae *cells were washed away easily. It is not known whether *K. pneumoniae *cells produce polysaccharide which adds/facilitates adsorption of cells to the surface of bonechar or other surfaces. Even though *K. pneumoniae *cells do not form firm layers of cells, these reactors are still highly productive.

**Table 6:** A comparison of 2,3-butanediol productivity in a packed bed biofilm reactor with productivities in other reactor types

**Reactor Type**	**Culture**	**Substrate**	**Productivity [gL^-1^h^-1^]**	**Reference**
Biofilm, continuous	*K. pneumoniae*	Whey permeate	11.70	[23]
Batch (control)	*A. aerogenes*	Glucose	1.10	[96]
Continuous reactor (free cells)	*K. pneumoniae*	Glucose	4.25	[97]
Immobilized cell continuous	*K. pneumoniae*	Whey permeate	2.30	[98]
Cell recycle, continuous	*B. polymyxa*	Whey permeate	1.04	[95]
Cell recycle, continuous	*K. pneumoniae*	Glucose	9.84	[99]
Cell recycle, continuous	*E. aerogenes*	Glucose	5.40	[100]

### Production of other chemicals in biofilm reactors

Other examples of production of industrial chemicals produced in biofilm reactors include acetic acid or vinegar, lactic acid, succinic acid, and fumaric acid. Acetic acid production in trickling bed biofilm reactors is a mature technology and is exercised at the commercial level [13]. In addition to trickling bed biofilm reactor, a submerged process was also developed in late 1940s. The acetic acid is produced by one of the bacteria grouped in the two genera, *Gluconobacter *and *Acetobacter*. The species that are used commercially include *Acetobacter aceti*, *A. pasteurianus*, and *Gluconobacter oxydans*. In the trickling bed biofilm reactor (volume 60 m^3^), beechwood shavings are packed and the starting material (alcohol solution) is sprayed over the surface. To this solution, initially, nutrients and bacteria are added for growth of biofilm on the beechwood shavings. The liquid trickles to the bottom of the reactor containing acetic acid. In order to increase concentration of acetic acid, the liquid is cooled and pumped back to the top of the reactor. Of the alcohol added, approximately 90% is converted to acetic acid during the trickling process. Approximately 120 gL^-1 ^acetic acid is obtained in 72 h, thus resulting in a productivity of 1.67 gL^-1^h^-1^.

Production of lactic acid in biofilm reactors is another example of industrial chemical production in such reactors. Demirci et al. [101] evaluated a number of supports for biofilm formation using lactic acid producing cultures. It has been reported that the best biofilms were obtained with *Pseudomonas fragi*, *Streptomyces viridosporus*, and *Thermoactinomyces vulgaris *when used in combination with polypropylene composite chips. The polypropylene composite chips contained polypropylene and 25% (w/w) agricultural material. The mixture of these components was extruded through an extruder to form chips of desired dimensions. Following this, a number of reports appeared from the same group on synthesizing, evaluating, and using various supports for biofilm formation and lactic acid production [102-105]. In one of the reports [104], lactic acid was produced in repeated batch cultures in a biofilm reactor. The reactor productivity was improved from 2.78 to 4.26 gL^-1^h^-1^. A maximum lactic acid concentration of 60 gL^-1 ^was produced in biofilm reactors where plastic composite support was used for adsorption.

In a study on the production of lactic acid by adsorbed cells of *Rhizopus oryzae*, the culture was immobilized on a fibrous-bed and used in a bioreactor [37]. The fibrous bed was a sheet of 100% cotton cloth onto which the culture was adsorbed. In this reactor (fed-batch), a productivity of 2.5 gL^-1^h^-1 ^was obtained with a high yield of 90% and a high product concentration of 127 gL^-1^. Glucose was used as a substrate. When glucose was replaced with cornstarch, yield improved to 100% and productivity decreased to 1.65 gL^-1^h^-1^. Using starch as a substrate, a product concentration of 126 gL^-1 ^was achieved.

Other reports on using cell support for cell growth and lactic acid production are those of Park et al. [106] and Sun et al. [107]. Park et al. [106] used 3 gL^-1 ^mineral support (Aid-Plus; ML-50D, Mizusawa Chemical Co., Niigata, Japan) and 5 ppm polyethylene oxide to flocculate the culture and change mycelial morphology from a large pellet to mycelial flocs. Sun et al. [107] immobilized cells of *R. oryzae *in polyurethane foam cubes. There are other reports on the use of immobilized cell technology to produce lactic acid, however, they have not been mentioned either due to space limitation or studies are not directly related to biofilm formation.

Biofilm reactors have also been used successfully for the production of fumaric acid [108] and mineral ore treatment [13]. In an interesting study, Cao et al. [108] used plastic discs to adsorb cells of *R. oryzae *to produce fumaric acid from glucose. The use of the biofilm reactor resulted in an increase in reactor productivity from 0.9 gL^-1^h^-1 ^in a free cell stirred-tank reactor to 4.25 gL^-1^h^-1 ^in the biofilm reactor. In the latter reactor, fumaric acid concentration up to 85 gL^-1 ^was obtained from 100 gL^-1 ^glucose. The fermentation time was shorter and took 20 h as compared to 72 h in the free cell reactor.

Succinic acid is a chemical that has been produced in biofilm reactors. The industrial potential for succinic acid fermentation was recognized as early as the late 1970s [109]. Succinic acid (HOOCCH_2_CH_2_COOH) is a dicarboxylic acid, which can be used as a feedstock chemical for the production of high value products such as 1,4-butanediol, tetrahydrofuran, adipic acid, γ-butyrolactone, and n-methylpyrrolidone [109] for applications in agriculture, food, medicine, plastics, cosmetics, and textiles. In a recent study on succinic acid production using *Actinobacillus succinogenes*, Urbance et al. [110] employed the customized plastic composite support (PCS) [111] and 20 other different PCS blends with and without mineral salt additions and evaluated 20 simulated repeated-batch fermentations using MgCO_3 _for pH control and CO_2 _supply. The customized plastic composite support (PCS) blends were screened for biofilm formation and succinic acid production. Succinic acid concentrations, percentage yield of succinic acid, and biofilm formation for each PCS blend were determined and no correlation between biofilm formation and succinic acid production was observed. However, the customized PCS blend for *A. succinogenes *in succinic acid production demonstrated 70% yields for succinic acid compared to 64% yield for suspended cell bioreactor [110]. Table 7 shows production of various chemicals in biofilm reactors.

**Table 7:** Production of various other chemicals in biofilm reactors

**Product/Reactor Type**	**Adsorption support**	**Productivity [gL^-1^h^-1^]**	**Reference**
**Acetic acid**			
Trickling bed biofilm reactor	Beechwood shavings	1.67 (120)	[13]
**Lactic acid**			
Agitating continuous reactor	Fibrous bed (cloth)	2.5 (126)	[37]
**Fumaric acid**			
Rotary continuous reactor	Plastic discs	4.25 (85)	[108]
Strirred-tank (control)	None	0.91	[108]
**Succinic acid**			
Repeated batch fermentations	Plastic discs	-	[110]

Numbers in bracket – product concentration in gL^-1^

## Enhanced rates of production of chemicals in biofilm reactors

### Length of operation of biofilm reactors

Packed bed reactors often block due to excessive cell growth. It should be noted that reactor blockage depends on a number of factors including cell growth rate, packing density of the support, and supply of nutrients. This type of reactor has been operated ranging from 2 weeks to 3 months. Tyagi and Ghose [4] used a packed bed biofilm reactor of *S. cerevisiae *for a period of 35 days, while Qureshi et al. [87] used a packed bed biofilm reactor of *E. coli *for a period of 103 days for ethanol production in continuous operation. However, it was observed that packed bed biofilm reactors of *C. acetobutylicum/C. beijerinckii *blocked sooner than 103 days due to enhanced cell growth of these cultures. In order to prolong life of the reactor, feed media deficient in nutrients should be attempted as used by Qureshi & Maddox [6] and Qureshi et al. [90]. It has been observed that this type of reactor blocks at the bottom where fresh feed allows excessive cell growth. In the upper part of the reactor, minimal growth occurs due to product inhibition as in case of butanol and ethanol production. In such cases, inverting the reactor can prolong life of reactor. In addition to the reactor blockage due to excessive growth, influent to the reactor plays an important role in prolonging life of the reactor. Reactor feed may contain suspended and particulate solids, in particular with wastewater influents, which may block the reactor. It is suggested that such influents be filtered or centrifuged to remove suspended and particulate solids to prolong reactor's life.

Fluidized bed reactors do not block due to excessive growth and they can be operated for a long period of time (>4 months). It is also viewed that UASB and EGSB can be operated for long periods. Table 8 shows length of operation of different reactors for the production of various chemicals, their productivities and dilution rates. Biofilm reactors are highly productive as compared to other reactor systems. The reader is advised to refer to the production of various chemicals in biofilm reactor systems (in this article) to be able to compare their production rates with the other non-biofilm reactor systems.

**Table 8:** Length of operation of various biofilm reactors used for the production of different chemicals

**Chemical Produced**	**Reactor Type**	**Length of operation [Days]**	**Dilution rate [h^-1^] (Productivity [gL^-1^h^-1^)**	**Reference**
Butanol	Packed bed	61 days	0.30–1.00 (0.98–4.10)	[6]
	Fluidized bed	>4 months	0.33–1.37 (1.65–5.10)	Unpublished data^1^
Lactic acid	Various reactors	Reviewed in ref 79 (Table 1)	- -	[37]
Ethanol	Packed bed	35 days	0.12–0.48 (7.80–28.60)	[4]
	Packed bed	103 days	0.04–0.12 (1.10–2.58)	[87]
	Packed bed	60 days	0.50–5.76 (5.00–74.88)	[86]

^1 ^Qureshi & Maddox

- Not reported

### Barriers in biofilm reactors

In adsorbed cell biofilm reactors of *C. acetobutylicum*, it was identified that there were four different cell types: growing cells, butanol producing cells, dead cells, and inactive cells (non-growing, nutrient requiring) [7]. Cells that were involved in butanol production were only a fraction of the total cells. For example, the concentration of cells in the reactor was approximately 74 gL^-1^, while the butanol producing cell mass was <10% of the total cells. The amount of dead cells or spores occupied most of the space in the reactor. It is viewed that if sporulation is blocked, the reactor productivity could be increased by many fold. This would improve the process economics of butanol production in biofilm reactors. At this stage we are not aware if this is applicable to the other organisms such as ethanol, 2,3-butanediol, succinic, acetic (vinegar), lactic and fumaric acid producers. It is suggested that this be investigated for the cultures that produce these chemicals. In UASB internal mixing is not optimal which reduces efficiency of the reactor [11]. This produces dead space in the reactor. For that reason expanded granular sludge bed (EGSB) are investigated [11].

### Diffusion limitations

Usually biofilms contain multiple layers of cells. The thickness of the biofilm may vary from a few to many μm. An increase in the biofilm particle diameter affects hydrodynamic conditions in the reactor including fluidization characteristics etc [59]. In order to measure the thickness of biofilm in *C. acetobutylicum *culture (PBR), an electron transmission micrograph was taken of a particle and it was identified that the biofilm was made up of >30 cell layers (Fig. 3c). In order for the cells to be active and be taking part in the reaction, nutrients and substrate must diffuse/penetrate to the inner layers of cells. However, it is likely that the nutrients and substrate are used up by the outer cell layers before they reach the innermost cell layers. If this is true, the innermost layers would neither survive nor take part in the reaction. Another example where the thickness of cell layers is an important consideration is bioparticles in a fluidized bed reactor. In these reactors the size of the bioparticles is much bigger than the bioparticle in PBR and cell layers are >>30. Accumulation of so many cell layers adds to the diffusion resistance to the substrate and nutrients. In order to keep the diffusion resistance to a minimum possible level, the size of the bioparticle should be kept to a minimum level while still keeping productivity of the reactor high. This should increase the rate of reaction and benefit the process economics.

In aerobic biofilm processes, such as oxidative degradation of toxic chemicals and production of acetic acid in trickling bed biofilm reactors, a constant supply of oxygen is essential. The oxygen should be dissolved in the liquid and be transported to the innermost layers. The penetration depth of oxygen should be 100% of the biofilm thickness. If the bioparticle size is large, then the inner layers would be starved of oxygen and the cells would die thus decreasing the conversion efficiency of the process. Supply of oxygen rather than air to the reactor would improve diffusion of oxygen to the inner layers; however, it would add to the cost of the process. Hence, size of the biofilms should also be kept to low to keep the reactor productive. In aerobic wastewater biofilm reactors oxygen is an important substrate/nutrient [12]. For anaerobic systems oxygen is toxic.

In addition to the above limitations, an additional limitation comes from the toxicity of product/s itself. Many of the fermentation products are toxic to the cells that produce them. Examples of such toxic products are those that have been described in the earlier section of this article. Butanol is toxic to the cells of *C. acetobutylicum/C. beijerinckii *and at higher concentrations it kills the cells. In the biofilm layers, the diffused substrate is converted to the products such as butanol. It is not known how quickly the produced butanol diffuses out of the cell layers. It is conceivable that accumulated butanol or other chemicals kill the cells before it is diffused out. It is also likely that a combination of nutrient deficiency and toxicity affects the cells more adversely.

## Industrial/pilot-plant level biofilm reactors

### Wastewater treatment

**Figure 6:**
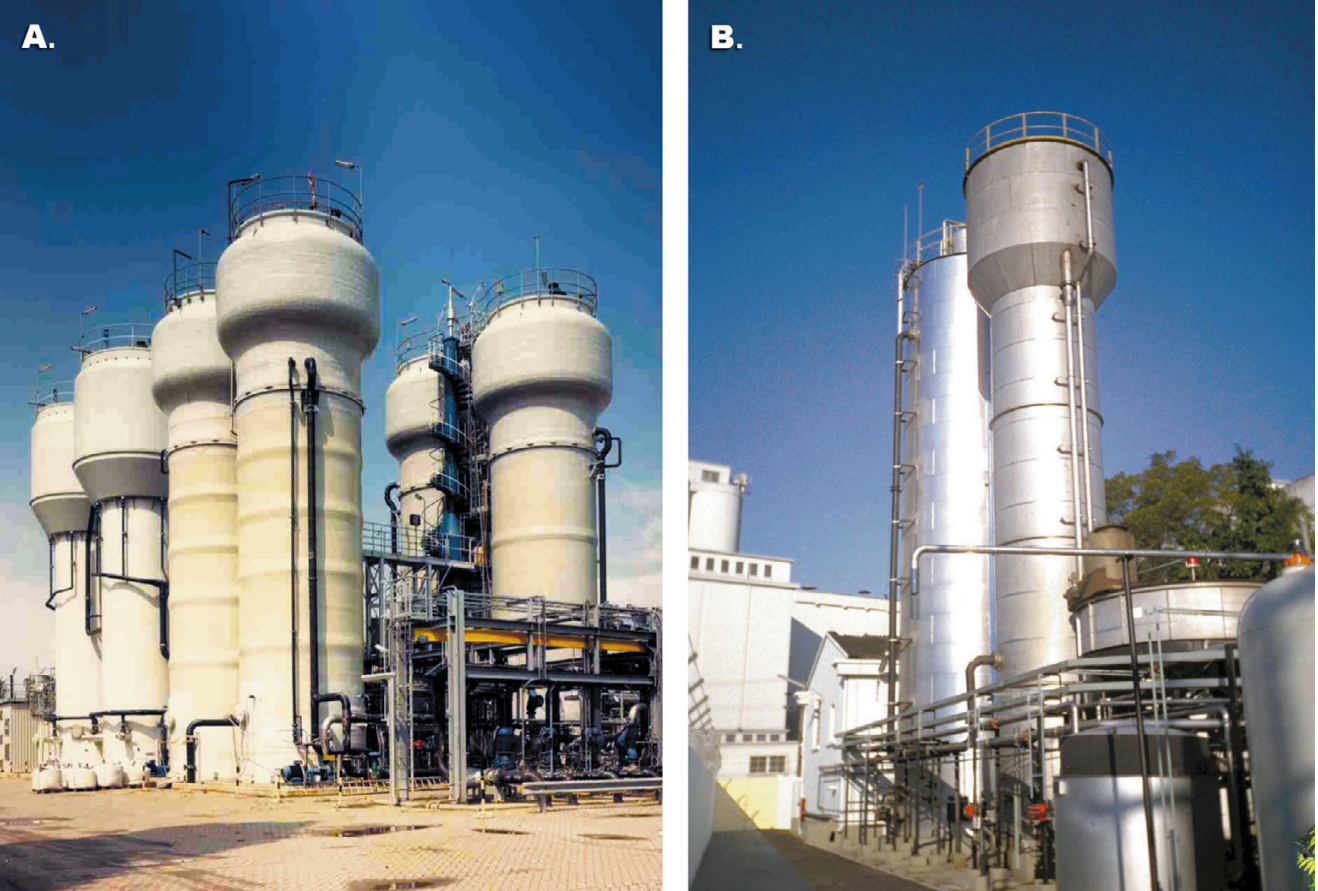
Full scale biofilm reactors: **(a) **biothane biofilm airlift suspension and expanded granular sludge blanket (Biobed) reactors at Gist Brocades, Delft (The Netherlands); **(b) **Pagues CIRCOX (foreground; 140 m^3^) and internal circulation (background; 385 m^3^) reactors at a brewery in Brazil. Reprinted from "Nicolella C, van Loosdrecht MCM, Heijnen SJ: **Particle-based biofilm reactor technology. ***Trends in Biotechnology *2000, **18: **312–320, with permission from Elsevier, United Kingdom.

Biofilm reactors have successfully been used in wastewater treatment [9-12,43,59,61]. In these industrial biofilm reactors cell mass concentration as high as 30–40 gL^-1 ^could be maintained [12,58]. As a result of superior efficiency, biofilm reactors are being used throughout the world with a number of full scale application for industrial and wastewater treatment. Examples of these reactors operating in The Netherlands and Brazil are shown in Fig. 6.

### Acetic acid/vinegar production

Commercial production of acetic acid or vinegar using biofilm reactors has been exercised for many years. Production of these chemicals has been reported by Crueger & Crueger [13]. Large biofilm fermentors of size up to 60,000 L have been used. Often beechwood shavings are used as a support for biofilm formation. For this system, trickling bed reactors have been used with an exit product concentration up to 120 gL^-1 ^and a productivity of 1.67 gL^-1^h^-1^. A description of the process has been given in previous sections.

### Butanol production

Butanol production in biofilm reactors has been practiced in numerous types of reactors at laboratory scale [6,38,39,44] with superior productivity to batch, fed-batch, and free cell continuous fermentations. Two of the most prominently used reactors are packed bed and fluidized bed reactors. In these reactors, productivities of the order of 4.5–15.8 gL^-1^h^-1 ^have been achieved as compared to productivities of 0.10–0.38 gL^-1^h^-1 ^in batch reactors. Given the scenario of increasing petroleum prices, it is suggested that fluidized bed reactors be scaled up to pilot plant level in view to further commercialize this fermentation.

### Other processes

Production of other industrial chemicals such as lactic acid and 2,3-butanediol should be exercised at pilot plant level. Nicolella et al. [12] reported that biofilm reactors are in operation at industrial scale throughout the world. Use of biofilm reactors is anticipated to be economical for the production of these industrial chemicals.

## Future directions & conclusions

A comparison of biofilm reactors with other reactor systems suggests that biofilm reactors are simple and offer higher productivities than other reactor systems. In biofilm reactors, cells can be adsorbed within the reactor without the use of any chemicals, and the reactors can be operated for long period of times. This would help in reducing the process cost. These reactors are already in use for wastewater treatment and acetic acid/vinegar production by fermentation. It is clear that their use at bench scale has been consistently increasing for the production of various other chemicals. As productivities in these simple biofilm reactors are high, their full potential should be employed for biotechnological/biological conversion processes. For further reading on biofilms and their formation, the reader is referred to the comprehensive articles published by Costerton et al., [112] and O'Toole et al., [113].

## Authors' contributions

NQ would like not to mention the contributions made by individual authors. However, it is stated that all the authors made significant contributions to deserve to be contributing authors of this comprehensive article on "Biofilm Reactors."

## Note

** Mention of trade names of commercial products in this article is solely for the purpose of providing scientific information and does not imply recommendation or endorsement by the United States Department of Agriculture.
